# Is Radiography Ready for More Assistant Practitioners? A Qualitative Case Study Approach to Examine the Contemporary Role in Diagnostic Imaging Services

**DOI:** 10.2147/JMDH.S605803

**Published:** 2026-09-01

**Authors:** Beverly Snaith, Sarah Etty, Robert Appleyard, Julie Nightingale

**Affiliations:** 1 Faculty of Health & Social Care, University of Bradford, Bradford, UK; 2 Radiology, Mid Yorkshire Teaching NHS Trust, Wakefield, UK; 3 School of Health & Social Care, Sheffield Hallam University, Sheffield, UK

**Keywords:** assistant practitioner, radiography, diagnostic imaging, allied health, skill mix, apprenticeship

## Abstract

**Introduction:**

The assistant practitioner (AP) role was introduced into the United Kingdom radiography profession to grow diagnostic imaging capacity and enable radiographers to take on more complex tasks. Despite limited evaluation into the utilization and impact of the role, recent national workforce strategies have called for further expansion of AP numbers.

**Purpose:**

To examine the contemporary status of the AP within imaging departments, identifying how and why roles have been established, the benefits and challenges of their deployment and potential for further expansion of this workforce group.

**Methods:**

Forming part of a wider mixed methods explanatory study, this qualitative research utilized a case study approach. The individual cases represented nine geographically diverse English NHS imaging departments. Data collection took place during single day visits and comprised multiple interviews and focus groups including APs (n = 30) and service leads (n = 34) together with collation of AP specific role documents. Within and across case analysis was undertaken.

**Results:**

The assistant practice level expands the healthcare workforce and increases clinical capacity. Whilst some imaging services have embraced the role, others are reticent. The reasons appear multifactorial including an unclear scope of practice, lack of standardized education pathway or curriculum and limited career progression opportunities.

**Conclusion:**

Twenty-five years after its inception in the radiography professional landscape, the AP role is embedded in imaging departments, but not at the scale originally envisaged. There remains a clear potential to utilize the AP workforce to expand capacity in the diagnostic imaging sector.

## Introduction

Ensuring the supply of an appropriately educated workforce is one of the biggest challenges facing healthcare providers. In the United Kingdom (UK), the National Health Service (NHS) is the largest employer,^1^,^2^ and therefore maintaining sufficient levels of staff with the relevant skills and experience is a critical and challenging process.^3–6^ The effects of the COVID-19 pandemic are still being felt in the NHS with waiting lists at an all-time high,^7^ and staff experience, well-being and retention adversely affected.^8^ One key area facing this challenge is diagnostic imaging, with persistently long waiting lists influenced not only by the pandemic but also increasing demand from technological developments and an aging population.^6^,^8^,^9^ Expansion of cancer screening and expectations of care closer to home have led to the development of additional community diagnostic centres,^9–12^ placing increasing pressure on already stretched resources.

Shortages in the imaging workforce are a global problem, impacting both medical (radiology)^13–16^ and allied health (radiography)^17^,^18^ professions. As a result, many healthcare systems have turned to international recruitment to address staffing deficits,^18–23^ although recent UK health workforce policy signals plans to reduce reliance on immigration over the next decade.^24^ The current workforce crisis is not new; the UK faced similar challenges at the end of the last century, and at that time proposed a long term solution through greater skills mix.^25^ To release radiographer time to take on more complex duties previously undertaken by radiologists, an assistant practitioner (AP) tier of practice was introduced. This development aimed to increase imaging capacity and grow radiographer numbers by introducing a career progression pathway for the imaging support workforce.^26^ This UK strategy is reflected in the support and assistive levels of practice internationally across professions, where this new role may be referred to as “assistant”, “technical instructor” or “technician”, although there is recognised variation in role content.^27^

Following the introduction of the AP role, there has been limited scrutiny or evaluation, and instead a greater focus on the higher levels of practice.^27^,^28^ But in 2020 a national review of diagnostic services set out an ambitious plan to train 4,000 additional radiographers and 2,500 APs by 2025, alongside the expansion of the advanced practice and radiologist workforce.^29^ The recommendation for this dramatic increase in numbers of APs was perhaps surprising given the lack of evidence of their impact.^27^,^28^,^30–32^

With limited contemporary knowledge on the integration of the AP role into imaging departments,^28^ this article examines the contemporary status of the role within imaging services in England. Situated within a wider project examining the support and assistant practitioner workforce in diagnostic imaging,^33^ the research aims to clarify the level of role establishment together with the benefits and challenges of their deployment. It examines the perspectives of both established and trainee APs, and their supervisors and managers to establish the context in which the role operates and consider the potential for its expansion.

## Background

To understand the AP development within UK diagnostic imaging, it is important to position their role preparation in comparison with the wider diagnostic radiography profession. Historically, radiographer education was delivered within hospital-based schools with a national diploma examination set by the professional body.^34^,^35^ The move into higher education and degree qualification in the 1990s changed the status of the hospitals from training centers to clinical placement providers. However, the more recent introduction of a parallel degree apprenticeship pathway to practice in 2019 has provided an opportunity for hospital staff to be employed as an apprentice radiographer (AR) whilst undertaking on the job training and academic study for their degree.^36^

**Figure 1:**
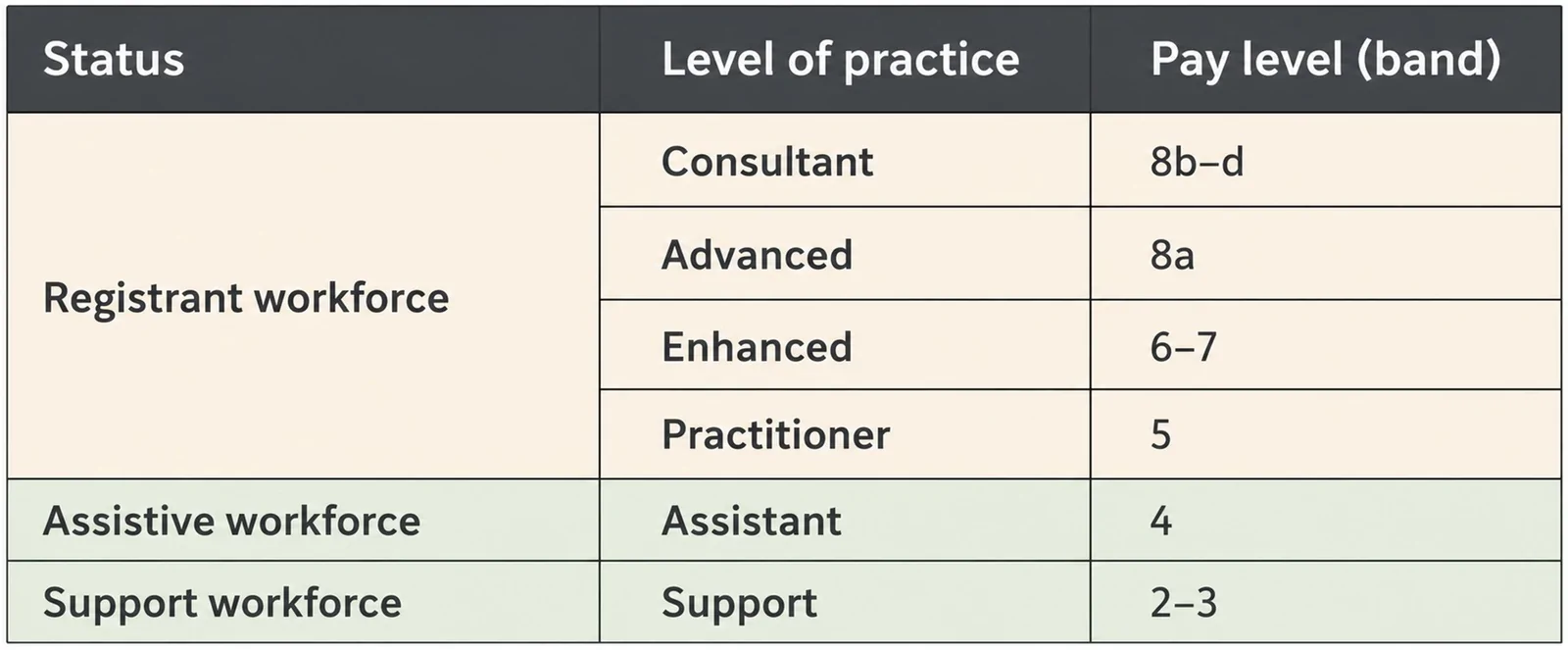
The levels of practice within the NHS non-medical workforce.

The AP tier of practice was developed to respond initially to meet the needs of a new national breast screening programme, supporting radiographers to undertake further postgraduate education to support role development.^37^ Initially recruited from imaging dark room technicians and support workers (SWs), APs are situated within a single area of practice eg mammography (breast imaging) or X-ray imaging, and work under the supervision of a radiographer.^28^,^38^ With interval iterations, this assistive tier now forms part of the wider non-medical NHS workforce (Figure 1).

Unlike their radiographer colleagues, AP training pathways do not specify the route, qualification or academic content.^31^,^39^,^40^ In the absence of a formal curriculum, guidance states that their “qualification should provide equivalent underpinning knowledge as that of a radiographer performing the same examinations”.^41^ Courses have often been developed with education provider viability in mind and support the core generic requirements of APs across professions, with limited specialty content.^42^ The trainee AP is employed, usually full time, undertaking the clinical education component within their workplace.^43^ As such, they work alongside pre-registration radiography students and, additionally, the new generation of ARs. At many sites, practice educators (PE) support all students and trainees in the workplace, with others also contributing to practice-based supervision. Although the AP role is seen by some support workers as a career pinnacle, for others it is a vocational pathway into a professional education programme.^32^,^44^

## Methods

This research formed the final qualitative work package of a multiphase explanatory mixed methods project examining the imaging support worker and assistant practitioner (ISWAP) workforce across England.^33^ It employed a collective case study appoach^45^ to gain an in-depth understanding of the topic in a real-world context. Based on a previous research phase, 137 NHS Trusts had been categorised as high, medium or low employers of ISWAP as a proportion of their whole radiographic workforce.^46^ Nine NHS Trust “cases” were purposively selected with organizations drawn from each of the three employer categories, having a broad geographic spread and varying in size and complexity (Table 1). Outcomes from this research phase related to the role of the ISWAP workforce in operational efficiency^44^ and the specific factors affecting support workers^47^ have been published previously.

**Table 1:** Overview of the Case Study Sites and Data Collection

Site	Category	Setting	Qualitative Data Collection	Document Analysis
1	High	Medium size service, coastal. 1 main and multiple community sites	1 tAP focus group (n = 5) 1 interview with AP (n = 1) 3 interviews with SLs (n = 4)	AP JD/PS (n = 1) AP preceptorship pathway (n = 1)
2	High	Large size service, coastal. 1 main and a satellite site	1 focus group with AP/AR (n = 2) 1 interview with AP (n = 1) 5 interviews with SLs (n = 5)	–
3	High	Medium sized service, city/rural. 1 main and multiple community sites	1 AP focus group (n = 5) 4 interviews with SLs (n = 4)	Apprentice AP JD/PS (n = 2) AP JD/PS (n = 2)
4	Medium	Small sized service, city. 1 main and multiple community sites	2 interviews with managerial staff (n = 2) 2 interviews with SLs (n = 2)	–
5	Medium	Large sized service, city. 2 main and 2 community sites	1 interview with an AP (n = 1) 3 interviews with SLs (n = 3)	–
6	Medium	Large sized service, city. 2 main and 1 community site	1 AP/tAP focus group (n = 7) 1 interview with PE (n = 1) 1 interview with SL (n = 1)	Apprentice AP JD/PS (n = 1) AP JD/PS (n = 1) Apprentice AP induction (n = 1) AP scope of practice (n = 1)
7	Low	Large sized service, city. 1 main and 4 satellite sites	1 AP/AR focus group (n = 6) 1 interview with PE (n = 1) 4 interviews with SLs (n = 4)	Apprentice AP JD/PS (n = 1) AP JD/PS (n = 1) AP training syllabus (n=1) AP scope of practice (n = 2)
8	Low	Medium sized service, coastal. 1 main and 3 satellite sites	1 AP/tAP focus group (n = 2) 3 interviews with SLs (n = 3)	Apprentice AP JD/PS (n = 1) AP JD & PS (n = 1)
9	Low	Medium sized service, coastal/rural. 1 main site.	1 interview with manager (n = 1) 3 interviews with SLs (n = 3)	–

**Notes**: Category indicates the proportion of support workers/assistant practitioners to the whole radiographic workforce (High = approx 30%; Medium = approx 20%; Low = approx 10%).

**Abbreviations**: AP, assistant practitioner; AR, apprentice radiographer; PE, Practice Educator; SL, service lead; JD, job description; PS, person specification; tAP, trainee AP.

Ethical approval [222/HRA/4272] and NHS site permissions were in place, and an initial approach was made to the imaging manager at each site to clarify the study objectives. Participant information sheets were shared at this time with a clear message that involvement in the study was voluntary, and no demographic information beyond role title would be collected. Site visits took place between April and September 2024 with at least two members of the research team attending on a single day. The researchers included individuals positioned within and outside the field of study, including two diagnostic radiographers (BS and JN) with extensive experience in mixed methods research, workforce development and evaluation. To complement their subject specific knowledge and provide an independent objective perspective, the other researchers have an oncology (RA) and psychology (SE) background, both with significant postdoctoral research experience.

Staff focus groups and individual interviews were conducted predominantly face-to‑face in a designated location on the hospital site, although a small number were completed online due to staff availability. Focus groups were held with support workers and assistant practitioners, with the assistive workforce (n = 30) comprising trainee and qualified APs and, at several sites, ARs at the request of the clinical teams (Table 1). Semi-structured interviews were undertaken with managers; service leads and practice educators. Topic guides were developed by the researchers based on previous literature reviews^27^,^28^ and research phases. The focus group questions focused on recruitment, training, perceptions of their role, team structure, career progression, and future aspirations. Interviews with staff in leadership roles explored strategic ISWAP workforce planning, operational challenges, their role across imaging services, delegation of tasks, training and supervision, barriers and facilitators to SWAP utilization and their perspectives on skills mix. The interviews and focus groups were digitally recorded and transcribed verbatim by an independent transcription service. All participants provided written informed consent, and this was confirmed verbally before recording commencement. Field notes were made by the researchers and debrief meetings took place after each site visit enabling nuances related to organizational, professional or analytic perspectives to be drawn, an important step in reflexivity.^48^,^49^ In addition, role related documents including scope of practice, training plans, job descriptions (JDs) and person specifications (PSs) were received from a number of the sites (Table 1).

The transcripts (n = 54) were checked for accuracy and uploaded into Quirkos™ to support analysis. Following the methods of Braun and Clarke,^49^ two researchers (BS and SE) reviewed the data independently and collaboratively agreed a coding framework based on the initial review of one site. Regular whole team meetings ensured consistency in coding and additional categorization of codes by relevance to level of practice (SW and/or AP) and participant type was also performed within the platform. Coding continued until all transcripts were analyzed despite reaching data saturation.

For this secondary analysis, the data were exported to Excel for within and cross case analysis of the codes categorized as relevant to AP, using a framework approach.^50^ Key themes were generated, and participant quotes have been reported verbatim including the site code and participant role.

In addition, document analysis of the JDs, PSs and other site-specific role materials was undertaken by a single researcher (BS) using a priori themes related to role title and level, qualifications, supervision clinical responsibilities, attributes and skills. Again, verbatim extracts from the documents have been labeled by site code.

## Results

### AP Deployment

APs were not employed at all sites and where they were they commonly numbered only single figures with marked variation in deployment. Most commonly utilized roles were seen in X-ray, breast imaging (mammography), and magnetic resonance imaging (MRI), and only limited numbers across other imaging areas.

The justification given by managers for the introduction of the AP role was to supplement the radiographer workforce (Table 2). This was either in response to a high number of vacancies, or to increase overall capacity as *it frees up a qualified imaging member of staff* (NM Lead Site 7). One trainee AP stated that their role should enable the radiographers to take on greater responsibilities, but *it does not seem to be that way, I feel like we are all just doing exactly the same* (Site 1). A manager also reflected that the radiographer role had not developed *even though we have got the APs available (Site 1)*. Overall, variations in AP role content and scope were evident (Table 2). Many suggested that the role limitations such as the narrow scope of practice hindered their impact, and this could be addressed if their scope was expanded, particularly into fluoroscopy (dynamic X-ray procedures) in the operating theatres.

**Table 2:** Themes Related to AP Role Deployment

Theme	Sub-Theme	Indicative Quotes	Participant
Justification for the AP role	High vacancy rates	*We ended up a few years ago developing an AP role because we were low on staff, we are struggling to recruit*	NM lead - Site 7
		*we are really struggling to find qualified mammographers. and the training takes 12 to 18 months to do. Assistant practitioners, the training is very similar; however, they are easier to train. They are more readily available*	Breast lead - Site 3
	Enabling radiographer role development	*Increasing our workforce at the time, it did allow radiographers then to move on to more advanced role*	X-ray lead - Site 2
		*In order for them [radiographers] to do those [reporting] roles, there’s stuff that needs doing in the background as well*	NM lead - Site 8
Opportunity	Extending the scope of practice	*We’d really like to be able to get to a point where maybe they could work in an operating theatre, how can we enable that for them?*	PE - Site 7
		*We are just, at the moment, getting a document approved so our assistant practitioners can. approve the X-ray request. That will be like minor injury stuff in ED.*	X-ray lead - Site 2
		*I know I have been told about some APs undertaking fluoro [fluoroscopy] under supervision. That was looked at way back when I was doing it, but it did not get brought in*	PE – Site 6
	New service implementation	*In our community sites.we will have one radiographer and one AP [in X-ray]. Whereas if they were not there, it would be likely that there would be two radiographers*	tAP - Site 1
		*He [the AP] works on as a, predominantly non-contrast CT scanner … well not totally independently, he can press the radiation button with a supervised radiographer*	CT lead - Site 7
		*we have got an extremity [MRI] scanner that only does arms and legs*	CT/MRI lead - Site 2
Service limitations	Workforce size	*If we release roles. to have assistant practitioners, then we do not have enough money to actually employ enough radiographers to cover the nights.*	Manager - Site 9
		*If you have too many [APs] you are limiting your workforce, in that they cannot work outside of their scope of practice, and then if we are really short-staffed, the majority of your staff end up being assistant practitioners, puts a lot of pressure on the radiographers.*	X-ray lead - Site 2
	External (private) providers	*We took on a mobile MRI service [contracted external provider] which basically we shipped all our routine easy things out*	MRI lead – Site 4
	Clinical risk	*Think it is tricky with CT, just with the high dose*	CT lead – Site 1
		*I cannot see where the limited scope would fit [in CT]*	CT/MRI lead – Site 2

**Note**: AP, assistant practitioner; NM, nuclear medicine; PE, practice educator; tAP, trainee AP; CT, computed tomography; MRI, magnetic resonance imaging.

Proactive workforce planning was evident in some instances, particularly related to new equipment installations or service developments (Table 2). Examples included the introduction of computed tomography (CT) scanning as part of the national roll out of lung cancer screening (Site 7) and a separate installation of an MRI extremity scanner (Site 2); both scenarios include predictable and repetitive work. In other sites, the AP role across such imaging modalities was limited, with one lead radiographer stating that *in the general department [X-ray] and nuclear medicine they have AP roles, we do not in CT and MRI at this moment in time* (Site 5). The complexity of these areas and high radiation dose of CT was considered to present a risk some managers were not willing to take. Additionally, wider service changes were acknowledged to have negatively influenced the potential for AP roles, particularly where less complex work had been subcontracted to the independent sector to reduce waiting lists.

### Supervision

The APs were considered an additional member of staff but were unable to work independently.

The responsibility for ensuring the quality and standards of the episode of care therefore remains with the registered practitioner (Radiographer). The “episode of care” begins with the referral for imaging or treatment using ionizing radiation, and ends when the patient is discharged from the imaging department. (AP SOP – Site 6)

All JDs included a statement that the AP works under the supervision of a registered practitioner, with some clarifying that this may be *under direct or indirect supervision*. The concept of indirect supervision was defined in the SOP by one site as the *supervisor is aware of what they are doing but is not directly observing the task* (Site 6).

Although supervision by a radiographer was expected, with them retaining overall control of patient care, there were contradictions. In many of the JDs, the APs were a*ccountable* for their work, whether *high standard of clinical radiography* (Site 8), *assessing, planning, implementing and evaluating programmes of care* (Site 7) or *their actions* (Site 3). This implies the AP, as an unregulated member of staff working under the direction of a registered professional, had a degree of autonomy. There was further reference to their independent decision-making regarding reviewing image referrals once they had been qualified for at least three months.

An Assistant Practitioner may perform examinations without direct reference to a radiographer where the referral criteria fall fully within the local protocol document, the supervising radiographer is available and they have successfully been assessed as competent for this particular examination… the Assistant Practitioner will then be able to assess their own images... and direct the patient to the appropriate route for their results according to departmental protocols. (AP SOP – Site 7)

This “scope creep” was interesting, as it was a contributing factor in the decision not to continue the AP role within another site.

We had concerns raised that she [AP] was sending images to PACS prior to them being agreed, double-checked by a radiographer. So that supervision was a bit tricky (Manager – Site 9)

Further evidence of fluidity in the role was noted at a third site where an AP noted that *we are supposed to just stick to scope of practice, but we do not usually* (AP – Site 6). The APs did acknowledge their actions had implications for the supervising radiographer, but perhaps not the consequences with statements such as:

So it’s their licence I suppose that I could be tarnishing if I took those pictures wrong, which is why they might be a bit upset about that. (AP – Site 3)

### Education Provision

Service leads, PEs and APs reflected on how underpinning education is influential in the success or failure of the role (Table 3). Changes including introduction of the radiography degree apprenticeship appeared to have resulted in some sites moving away from employing APs to provide training capacity for ARs. Some senior staff members expressed their disappointment, reminiscing that *the old assistant practitioner literally sort of died a death* (Manager – Site 4).

**Table 3:** Themes Related to AP Education

Theme	Sub-Theme	Indicative Quotes	Participant
Education standards	Absence of national scope expectations	*[need] standardisation across the country, because that’s probably where it limits them as well in terms of their role development and ability to progress and move around, is what one AP does in one hospital is completely different to what an AP does in another hospital*	X-ray lead - Site 3
		*I am not aware of any sort of national standards for what assistant practitioners are under either an obligation to achieve themselves or what the employers are under an obligation to sort of teach them*	X-ray lead - Site 7
	Available standards	*We have sort of guidance from [NHS Breast] screening on what they can and cannot do and what their training should be*	Breast lead - Site 9
Education and training pathway	Different types of education providers	The three that we have trained did it through [FE] College, like a health sciences degree, and then it was the radiology specific stuff was done here. The two that are training currently are doing it with ****** University. It’s a specific assistant practitioner course. So that’s very different.	X-ray lead - Site 3
		*[we do] the first two years of the undergrad [undergraduate radiography degree] course basically. We do physics, anatomy, we do all the same things*	X-ray AP - Site 8
	Not imaging specific	*we’re actually thinking, look at those now, they’re doing three year apprenticeships that will actually cover the full degree, or potentially look at other places that do apprenticeships because they’ll be radiographic specific, rather than healthcare.*	PE – Site 6
	Variation	*We have done I guess like a new course compared to the other APs that have done it at a different college.*	Breast AP - Site 3
		We were waiting for the University of ***** to get theirs [degree apprentice radiographer course] online because they will run it in tandem with their undergrads … if we went with a completely different provider, it would be an absolute nightmare	Manager – Site 4
Apprentice	Benefits	*I think it’s a better way of producing a radiographer … they are less likely to leave the area because they have got links to the area. The person does not have to get into debt going to university and all these things, so they have got a joint interest*	AP - Site 3
		*I would not have been able to do that by quitting my job and going to uni, so it made it accessible, whereas it just would not have been an option otherwise. So I have kind of taken that hit with, it will be worth it in the end.*	AP - Site 6
	Challenges	*The levy covers the training but it does not backfill*	PE - Site 7
		*I have got to keep a post open. say that apprenticeship is three years or two years, that’s a long time to have a vacancy and not fill it*	X-ray lead - Site 1
		*We have not gone down the apprentice route yet. We have looked at it and to be fair we were waiting for it to stabilise a bit*	Manager - Site 4
	Entry criteria	*One of the blockers that I have noticed with other people is their maths and English*	tAP - Site 1
		*She would need to do some more maths and English, she did not have the grades to go on to do that*	NM lead - Site 8

**Abbreviations**: AP, assistant practitioner; FE, Further Education; PE, practice educator; tAP, trainee AP; NM, nuclear medicine.

AP training courses varied, with examples including a one-year certificate course from a local Further Education (FE) college and a two year foundation degree from a Higher Education Institute (HEI). Although these were considered equivalent within the reviewed PSs, the participants were not convinced. One AP (Site 5) reflected that they *never ever visited uni [university] on the AP course, it was all online*. This was exacerbated by the content of the HEI route being aligned to the radiography degree program whilst FE courses were more generic and expected the specialty-specific content to be taught by the NHS employer. This perceived increase in the expectations on the workplace was described by a PE (Site 7) who felt it was *the actual sort of nuts and bolts of their practice we are responsible for*. Others also reflected the need for greater communication between the education provider and employer.

massive miscommunication... work [hospital] doing what they can, and then they’re saying you’re going to get the rest over there [at HEI], and it just never matches up (tAP - Site 6)

I found that my AP course was very nurse-y, there was not much radiography stuff (X-Ray AP - Site 5)

Similar issues were identified in the initial iteration of the AP apprenticeship:

the first [AP] apprenticeship is quite inherently more generic healthcare, rather than as many radiographic specific modules as the undergrads [undergraduate students] have (PE – Site 6)

This was reflected in the PSs with some employers not specifying an imaging focused award, rather just reflecting the naming of their local training course.

Foundation degree in science, having followed an approved programme of study in Healthcare Practice (AP PS - Site 1)

Where it was specific to the imaging service, the level varied, although this was most apparent within mammography:

(Level 5) Radiography Assistant Practitioner Foundation Science Degree Qualification (AP PS -Site 6)

Mammography Associate Practitioner Level 4 or Foundation Degree of Science (FdSc in Health Sciences- mammo) or equivalent (AP PS – Site 3)

Many cited expectations in the training documentation related to the safe use of ionizing radiation within an imaging department and the national regulations.^51^

For the Assistant Practitioner to act as an [ionizing radiation] Operator... they must be entitled by their employer and be able to evidence “adequate education and training” for that role (AP SOP – Site 6)

Despite the proliferation of training courses, at one site some of the more established APs had received in-house training only and had not undertaken any academic qualification, thereby limiting their progression opportunities.

I think a weakness of that … we [radiographers] very much train them and train them very well to only work at [this hospital], it’s not transferrable (XR lead - Site 7)

we’ve sort of realized that actually they [APs] should really have the academic qualification, so that’s been our sort of mission since then to try and help them all to get on (PE - Site 7)

One site reviewed the assistive tier differently and considered those on the radiography degree apprenticeship to also be working as an AP level whilst studying.

just about to complete our first year at university to become full-time radiographers, firstly [assistant] practitioners, so I’m at the moment … we’re learning how to obviously scan MRI scans, then be signed off to be able to scan in there alone without supervision (AR – Site 2)

Trainees and qualified staff reflected the difference between the trainee APs employed by the hospital and the unpaid full-time undergraduate degree students attending placements.

because you’re [APs] here all the time, obviously the students they go away for three months. So, because we’re here the whole time we can get our standard of radiography … to a good level at an earlier rate than maybe the radiography students (AP – Site 3)

Although vocational in nature, fundamental differences were noted between the educational pathways. The apprenticeship was seen as an innovative route to a formal qualification as an AP or radiographer. For some, it was seen as *the only avenue for some of us, especially mature students* (AP - Site 8). The practicalities of the hospital-based paid training route were also attractive.

The BSc [degree] people get time, they get study days, they get one a week, they get free time and holidays. Whereas if we’re off uni [university] we work full-time instead, so I suppose that’s the sacrifice you make for being paid to do it (AP – Site 1)

we’re here all the time and we only spend a week in university three times a year, so everything’s on the job, which is brilliant (AR – Site 4)

Despite being employed as a “trainee”, individuals were still more likely to introduce themselves as a “student”, recognizing the challenges in defining their status.

I say student, but that’s probably just my choice of language (tAP - Site 1)

I’m in that limbo of being an apprenticeship where I’m not quite a student, but I’m not quite a radiographer, we’re in that middle bit (AP - Site 7)

### Recruitment and Reward

There were perceived challenges to the different training pathways, particularly as trainee APs and all apprentices were paid whilst training, although there was no standard salary. This was exacerbated when trainees were also expected to continue some of the responsibilities of their previous support worker role.

Whilst this role is primarily a clinical training role there will be a requirement to undertake some duties of an Imaging Assistant at the same B2 [band 2] grade (JD – Site 6)

Although many trainee APs were appointed internally from support worker or administrative roles, some hospitals had externally recruited but often expected *previous experience of working within a healthcare environment* (tAP – Site 6). Some organizations had a defined pay level for trainees and clear criteria. Whilst at other sites it was a case of *whatever band you were on for your initial [previous] job role, it just continued throughout your training post* (AP – Site 1). This introduced inequity between trainees but individuals accepted this situation, with reports that *people have taken pay cuts to come and do it* (tAP – Site 6). For those trainees undertaking the radiographer degree apprenticeship there was again inconsistency with one advertising the AR role as a NHS pay band 4 and another employing an acknowledgement of a step change in their pay related to their level of knowledge and skills.

We start at a band 3 for the first two years, the third year we’re paid as a band 4, the same as the APs, because we’re classed as APs, we’re the equivalent of AP then. (AR – Site 2)

### Career Progression

Some managers saw the AP role as an opportunity to create a workforce pipeline from support worker through to registered radiographer. A small number of sites had supported APs to step onto a degree pathway, a so-called “top up”. This was only available in a small number of sites and even then, there was no guarantee of the opportunity (Table 4).

**Table 4:** Opportunities for Workforce Development and the AP Role

Sub-Theme	Codes	Indicative Quotes	Participant
Proactive career progression	Aspirations	*If there’s a* *pathway to then become a* *radiographer, I* *think it would be a* *very sensible way to increase the amount of radiographers in the country*	AP - Site 3
		*My aim is if we keep putting a* *couple [of apprentices] in each* *year they will plop out the other end as radiographers after four years*	CT/MRI lead - Site 2
	Evidence of success	*We have got a* *senior radiographer in CT that was an AP, one of my clinical educators was an AP*	PE - Site 6
		*I* *have been thankful that someone was looking down on me and put me in the right position, so it’s been good for me for my future. For the hospital’s future I* *think it’s also a* *positive, because we are struggling to get radiographers*	AR - Site 2
	Staged approach (AP and then top up)	*We have currently got two APs that have, they could work as qualified APs but they are on the bridging course to become band 5 radiographers*	X-ray lead - Site 5
		*It’s not like after two years you are guaranteed the opportunity [to top up], it’s very much dependent on there being a* *post available.*	X-ray lead – Site 7

**Abbreviations**: AP, assistant practitioner; CT, computed tomography; MRI, magnetic resonance imaging; PE, practice educator; AR, apprentice radiographer.

Overall, there was a clear sense of a “bottleneck” in the career pathway, despite staff and manager desire for progression (Table 5). This was predominantly linked to financial pressures, but also the challenge of continuing academic study, often linked to the absence of a standardized curriculum. There was a perception that there was limited acceptance of prior academic (or experiential) learning, particularly where the AP role was outside of the well-established X-ray specialty. Additionally, there were acknowledged employment contract challenges of supporting a trainee AP who then transfers to a traditional student programme (top-up degree). This appeared to be informing a preference to only support the three-year degree apprenticeship.

[I] think the apprenticeship would do better if we maybe gear towards the three years, rather than the two [year AP apprenticeship] and then a break, then an 18 [month] or a two year [to degree level]. There can be difficulties changing their contracts all the time, because they’re temporary. (PE - site 6)

**Table 5:** Challenges to AP Progression and Implications for Workforce Planning

Sub-theme	Codes	Indicative Quotes	Participant
Limited opportunity	Lack of progression	*The four-tier structure and allowing people to move up through the tiers, I still feel that’s quite rare*	X-ray lead - Site 3
		*She [the AP] was angry, there was no progression. I do not think I would replace this job with another assistant practitioner until we have more clarity about the training and their career progression*	Breast lead - Site 9
	Bottleneck	*We had two support workers who applied for the apprentice post a couple of years ago, we had three internal candidates, but only one post*	Manager - Site 9
		*It’s not like after two years you are guaranteed the opportunity, it’s very much dependent on there being a post available.*	X-ray lead - Site 7
		*Our manager … she wants us to work a few years as a band 4 before bringing in some roles for the top-up*	AP - Site 8
		*If a band 4 assistant practitioner available came within my area then my whole training package would move because I am encouraging the [pay band] 2s to train as 3s, my 3 to train for my 4 and then eventually my 4 looking to leave me for a little while they went and did radiography to come back to me as I say. But I do not have that, it’s like a bottleneck.*	Breast lead - Site 7
		*It was like nine years before like any opportunities came up for an AP job.*	AP - Site 1
	Convoluted pathway	*… have to do an extra module to get onto the top-up, because we were not in X-ray first*	MRI AP - Site 6
		*I have to go and do plain film [X-ray] to be able to be a radiographer, so I’d have to go back to that, hopefully I’d be able to go back into MRI*	MRI AP - Site 3
		*I would need to be paying her for three years while she went and did her apprenticeship, and a lot of that time she would be away from us doing her placements in main radiology*	Breast lead - Site 9

**Abbreviations**: AP, assistant practitioner; MRI, magnetic resonance imaging.

Despite the challenges, there was clear evidence of job satisfaction, with many APs wishing to remain at this level of practice. Importantly, they felt valued within the wider team.

I don’t want to be a radiographer, I’m quite happy to stay as a band 4 AP (AP - Site 8)

I feel I’m respected for what I do (AP - Site 3)

## Discussion

This study of NHS imaging services in England found little evidence of the integration of the AP role into strategic workforce planning, despite it forming the bedrock of the career framework for the radiography profession and an ongoing national policy direction.^43^ Organizations that had implemented the role had primarily done so to create additional capacity and enable radiographers to take on more complex tasks, though evidence to support the latter is limited. Statements in the JDs implied all the studied AP roles were *assistive*^52^ with the supervising radiographer remaining responsible for episode of care. In practice, the APs themselves and their SOPs confirmed that they were more aligned to blended supportive/substitutive jobs.^52^ This mirrors the AP survey findings of Palmer et al,^43^ which identified that APs may complete all activities within a patient pathway from arrival to discharge, including acquiring and reviewing images without reference to a radiographer. This tension between service flexibility and workforce accountability is a point of debate.^52^ This is likely exacerbated by the extensive experience of some APs who may be significantly more established within their specialty than their supervisor. Munn et al in their systematic review examining the use of the assistive tier internationally also identified concern amongst health staff regarding their responsibilities for supervision.^53^ This blurring of boundaries was further emphasized within our study as both APs and radiographers were undertaking protocol-driven care with both defined as an “operator” under the national Ionizing Radiation (Medical Exposure) Regulations, rather than taking on the higher-level justification as a “practitioner”.^51^

Despite the acknowledged need to expand the imaging workforce and provide an alternative route to professional registration, there was clear reticence from those in leadership roles about the value of the AP role. Such concerns mirror those of the nursing profession about a similar role, the nursing associate (NA), first developed in 2017,^54^ partly as a result of concerns around care standards.^55^ The NA was also designed to bridge the gap between the nursing support workforce (healthcare assistants) and the registered nurse.^56^ Justification for the move of nursing away from the AP role and to a defined tier within the profession was the requirement for this level of practice to be able to administer medicines, a task only able to be performed by regulated professions.^57^ The key difference between the roles is that the AP title does not define a single profession and as such is not regulated.

There was evidence of heterogeneity in education provision often leading to “localized competence”, thus undermining the portability of skills. The AP role across the AHPs remains unregulated in the UK and therefore is not subject to the same curricular review or professional oversight as the NA, despite the same academic, and pay, level. In contrast, registered staff such as radiographers have clear expectations for the content of their approved pre-registration programmes. The inconsistency was a clear focus of concern to study participants, whether related to unclear course content or variation between providers. A number of AP education providers were noted to be aligned to radiography pre-registration programmes, and this was perceived as a benefit. However, in the context of NA education it is suggested that sharing of content with the student nurse can hinder the development of the NA professional identity.^58^ This is something which must be considered if the AP is not to be seen as a “cheap” solution to the workforce shortage and is to truly develop an identity as a role in its own right.

Aligned to previous studies,^29^,^32^ the assistant workforce explored in this study appears to predominantly, but not exclusively, emerge from the clinical support worker roles within the same healthcare setting. However, this created inequity as, similar to Thurgate and Griggs’ literature review, the AP trainees were often expected to provide workforce flexibility by fulfilling their previous duties during periods of clinical pressure, particularly as a consequence of staff shortages.^56^ Vocational training pathways are always subject to the ebbs and flows of service pressures, but this can lead to role ambiguity. The development route for support workers is seen as critical for workforce retention and brings a breadth of clinical skills and contextual understanding of the environment, however, it is important to develop a discrete level of practice, whilst providing flexibility to meet service delivery challenges.

Overall, there were low numbers of APs employed by the study sites, confirming the findings of Kessler and Nath in wider UK healthcare settings.^58^ Some participants felt that there were limits to the deployment of APs, particularly in certain settings including when the clinical radiation dose is high, something not seen in other AHP specialities.^27^ There is no evidence of any greater risk from introducing skills mix, although there are well-recognised incidents within medical radiation safety within the regulated professional groups.^59^ Some service leaders struggled to see how the limited scope would contribute to efficient service delivery in complex areas such as CT, correlating with the findings of previous research.^60^ Despite this, examples were identified where AP roles had been effectively implemented into these specialist areas.

AP roles within imaging have emerged as technology (modality) specialists because of the complex technologies used in each area and the safety critical processes involved in the use of ionizing radiation or high-strength magnetic fields. Although this mirrors the career path of radiographers,^61^ the AP lacks the cross-cutting educational preparation to move between modalities. This focus on specific services has hampered the progression of APs, exacerbated by inflexibility in education pathways. Radiography degree programmes have traditionally placed an emphasis on competence to perform X-ray procedures and therefore an AP in mammography, MRI or CT may not be perceived to have the broader knowledge and clinical skills to enable recognition of their prior learning. This lack of career progression was a source of concern for both APs and managers alike and mirrors previous research.^32^ However, this study identified that even when this career progression was supported, there was often a hiatus before enabling top-up training, perhaps designed to get value for money from the AP role.

One unanticipated impact on AP utilization was the parallel introduction of the radiography degree apprenticeship.^39^ The two vocational training routes (tAP and AR) have created competing pathways. Some NHS organizations have seemingly lost faith in the AP as a concept and instead have established the AR role. The AR scope and autonomy were considered by many employers to mirror that of the AP whilst training, as unlike undergraduate students the AR is employed as a member of staff. However, there are key differences in the education an AP has for their scope and level of responsibility within a specialized area, compared to the longitudinal three-year degree and its holistic imaging ethos. The contribution of degree apprenticeship trained radiographers to the new registrant workforce is growing, rising from around 3% in 2023 to over 13% in 2025, reflecting the maturing of the apprenticeship programme.^62^ It is as yet unclear whether wider introduction of AR posts will be the demise of the assistive tier in imaging, but both pathways also signal a return to hospital-based training with increased expectation on clinical staff to provide greater input into the education of trainees. The growth and diversification of apprenticeships and introduction of the national Levy as a tax on large employers^39^ requires in-service staff development with 20% off the job training. Despite this, many managers were reticent about the apprenticeship route, focused predominantly on the need to “hold” a substantive vacancy for the trainee to move into when qualified. The degree apprenticeship was designed as an alternative route to radiographer training, whereas the AP role was designed to undertake low complex imaging tasks to release radiographer capacity. Whilst the opportunity to utilize this as a progression path for APs is laudable, the expansion in the number of temporary inexperienced individuals working at the assistant level is contrary to the ambitions of the skills mix strategy. In contrast, the permanent AP role gives much more scope for building expertise over time and for developing their autonomy, a clear aspiration of some participants in our study. The risk of focusing on radiographer expansion, although no one denies the need, at the expense of the assistive tier risks losing these roles altogether. Longer term evaluations must consider how different policy drivers and academic developments impact the overall workforce profile. The national strategies for the English healthcare system rely on further expansion of diagnostic capacity.^11^,^12^ The ongoing focus on building community diagnostic centers to move non-complex work away from acute hospitals in theory provides an opportunity to tackle waiting lists but their cost-effectiveness will depend on staffing profiles.^10^ As such, APs may yet provide the skills mix aspirations outlined over two decades ago^25^ if their role, utilization and education are planned and executed effectively.

Several limitations to the research are acknowledged. Not all sites provided documentation regarding the AP role. Only nine sites were visited as part of the case studies, but the robust sampling process and common themes identified mitigates the risk of selection bias. Within the sites, due to workforce pressures on the day of site visits, there was variable representation from the different imaging specialities and therefore there are potential nuances not captured. Every effort was made to reduce the potential for researcher bias with at least one visiting researcher being from a non-radiographic background. Similarly, the bias within independent thematic analysis was mitigated through reflexive de-brief meetings following each site visit and analysis step.

## Conclusion

The assistant practitioner (AP) role was introduced into the UK radiography profession two decades ago to grow diagnostic imaging capacity and enable radiographers to take on more complex tasks. The contemporary AP role is now seen as a way of diversifying the registrant workforce, providing a new route to professional qualification through a vocational training pathway. As such, the potential for career progression is a key factor in the success of the role. The AP role is evident in many imaging departments, but not at the scale envisaged by the initial and recent national workforce strategies. Inconsistent scopes of practice, educational approaches and reticence amongst the registered workforce are seen as influential factors, as is a lack of evidence of their impact. This underlines the need for clearer standards for the assistive tier and a more cohesive approach to their training and deployment. Expanding AP numbers without this framework risks further inconsistency in deployment, weak portability and fragile role credibility.

There remains a clear potential to utilize the AP, and wider support workforce, to expand capacity in the diagnostic imaging sector, a driver which remains unchanged to date. While some centers value this role, there remains a dearth of evidence of workforce transformation through the utilization of the assistant level. The introduction of the degree apprenticeship into the radiography profession has had an unexpected impact on the AP role and it remains unclear whether the two routes are complementary or competitive. Strengthening coherence in the AP role requires improved understanding from the education provider and employer perspectives. Explicit curriculum and assessment expectations linked to a defined scope of practice, and more reliable workplace learning conditions (including protected time and supervision) are urgently required. Future research needs to go beyond the perception of roles and truly examine the impact of the skills mix initiative in relation to service capacity, efficiency and patient experience.

## Abbreviations

AHP, Allied Health Professional; AP, Assistant Practitioner; AR, Apprentice Radiographer; CT, Computed Tomography; FE, Further Education; HEI, Higher Education Institute; JD, Job Description; MRI; Magnetic Resonance Imaging; NHS, National Health Service; NM, Nuclear Medicine; NA, Nursing Associate; tAP, Trainee Assistant Practitioner; PS, Person Specification; PE, Practice Educator; SOP, Scope of Practice.
